# Assessing respiratory status in myasthenia gravis: limited value of the MG-ADL as a standalone tool compared with spirometry in a Danish cohort

**DOI:** 10.1007/s00415-026-13757-6

**Published:** 2026-03-24

**Authors:** Linda Kahr Andersen, Simone Birnbaum, Malene Missel, Kristine Grøsfjeld Petersen, Cas Mohringer, Eva Deurell, Nanna Witting, John Vissing

**Affiliations:** 1 Copenhagen Neuromuscular Center, Department of Neurology, Copenhagen University Hospital - Rigshospitalet, Inge Lehmanns Vej 8, 2100 Copenhagen, Denmark; 2 Institute of Myology, Paris, France; 3 Department of Thoracic Surgery, Copenhagen University Hospital - Rigshospitalet, Copenhagen, Denmark; 4 Department of Clinical Medicine, University of Copenhagen, Copenhagen, Denmark; 5 Amsterdam UMC Location University of Amsterdam, Rehabilitation Medicine, Amsterdam, the Netherlands

**Keywords:** Myasthenia gravis, Respiration, Respiratory dysfunction, Spirometry, Forced vital capacity

## Abstract

**Background:**

Patients with generalized Myasthenia Gravis (gMG) may develop respiratory involvement, which can be life-threatening. In clinical practice, the Myasthenia Gravis Activities of Daily Living scale (MG-ADL) is often used to assess patient-reported respiratory impairment, as spirometry can be time-consuming and require specialized equipment and expertise. However, it is unclear whether the MG-ADL is a sufficient screening tool for identifying respiratory insufficiency.

**Methods:**

In this cross-sectional study with a longitudinal subgroup, patients completed the MG-ADL, the Myasthenia Gravis Composite scale (MGC), and the Quantitative Myasthenia Gravis score (QMG). As a part of the QMG, spirometry was conducted. All patients had one evaluation, and a subgroup of patients had an additional evaluation after 3 years.

**Results:**

One hundred patients were screened, and 70 were included and tested. Of these, 25 were retested 3 years later. Respiratory symptoms were reported by 30 (43%) of the 70 patients, though none reported severe symptoms. There was a poor correlation (*r* = − 0.24, *p* = .048) between the respiratory item of the MG-ADL and spirometry. Patients with respiratory insufficiency showed improvement over time, as did the MGC score.

**Conclusions:**

Our findings indicate limited agreement between patient-reported respiratory symptoms (MG-ADL) and spirometry measures, suggesting that spirometry remains essential for accurate assessment of respiratory function in gMG.

## Introduction

Myasthenia gravis (MG) is an autoimmune neuromuscular disease characterized by fluctuating weakness and fatigue in skeletal muscles, resulting from autoantibodies targeting proteins of the neuromuscular junction [1]. In Denmark, the estimated number of individuals living with MG is around 1000 [2]. Approximately 425 of these are followed at the Copenhagen Neuromuscular Center (CNMC), a specialized neuromuscular outpatient clinic at Copenhagen University Hospital - Rigshospitalet [3].

Respiratory involvement may occur in patients with generalized MG (gMG) due to weakness and fatigue of respiratory muscles. Patients may experience shortness of breath during activities that are usually performed without difficulty, e.g., walking on a flat surface, and some also experience dyspnea at rest. These symptoms represent a severe manifestation of MG, as untreated respiratory involvement may progress to life-threatening deterioration, such as myasthenic crisis [4].

At CNMC, patients are routinely asked about respiratory symptoms once a year or every two years at their evaluation. They are advised to contact the clinic immediately if such symptoms occur at home. The clinician assesses the patient's symptoms and severity by one of the MG-specific scales: the Myasthenia Gravis Activities of Daily Living profile (MG-ADL) or the Myasthenia Gravis Composite scale (MGC), as an add-on to the anamnesis. As spirometry is time-consuming and demands equipment and skills, it is only performed if the medical history leads to suspicion of respiratory insufficiency. However, this approach requires that the patients are explicit about respiratory symptoms, especially as respiratory items are only a minor part of the MG-ADL and the MGC. Therefore, there might be a risk of missing serious respiratory symptoms in an outpatient setting.

The extent of respiratory insufficiency in gMG is sparsely reported [5–7], and more evidence is needed, especially on how to optimally recognize respiratory symptoms in an outpatient clinic. One study (*n* = 45) found no correlations between the MG-ADL/MGC and FVC [7]. However, this study included fewer, younger, and more female patients than the present study. In addition, the patient-reported MG severity was difficult to determine from the results. Another study found that FVC %predicted correlated weakly with clinical outcome measures in patients with facioscapulohumeral muscular dystrophy [8]. However, this study included no patient-reported symptoms.

This study aims to investigate respiratory status in a cohort of Danish patients with gMG. Specifically, we seek to:

- Assess respiratory status using both patient-reported questionnaires and spirometry.
- Evaluate if subjective respiratory symptoms correlate with spirometry measurements.
- Examine changes in respiratory status over time.

## Methods

### Individuals and procedures

This paper presents secondary analyses of data from two previous studies conducted at our clinic [3, 9], both focusing on clinical and functional assessments in patients with gMG. Participants were randomly recruited from the outpatient clinic at CNMC over the course of 8 months in the year 2019–2020 and completed questionnaires and tests, including spirometry [9]. Approximately 3 years later, patients from our clinic were again randomly recruited for another round of questionnaires and tests [3]. A subset of patients participated in both rounds of testing and therefore completed spirometry twice.

All participants were informed about the study either during their regular evaluation visits with their neurologist or by telephone. They were subsequently invited to a 1½-hour visit that included spirometry as part of the Quantitative Myasthenia Gravis score (QMG), the MG-ADL, and the MGC. In addition, baseline information about the patient and their disease was obtained. For participants treated with pyridostigmine, testing was scheduled 1½−2 h post-dose to ensure a stable medication effect. All assessments were performed by evaluators experienced in MG, who had trained together to ensure inter-rater consistency. Inclusion criteria were age ≥ 18 years and a confirmed diagnosis of gMG based on at least two of the following criteria: 1) clinical improvement with acetylcholinesterase inhibitor treatment, 2) presence of antibodies against the acetylcholine receptor, and/or 3) electrophysiological findings indicating abnormal neuromuscular transmission.

Exclusion criteria were comorbidities that could interfere with test results (e.g., pulmonary disease, dementia), pregnancy, or insufficient understanding of the Danish or English language.

To avoid including patients in full remission, participants with a combination of no MG medication and no MG symptoms were excluded.

The project was registered with the Danish Data Protection Agency (P-2021-828, P-2023-77). The Ethics Committee of Greater Copenhagen was contacted for approval. However, the committee exempted the study from regular approval due to its use of non-interventional tests routinely used in the clinic. Written and informed consent was obtained from all participants. The study conforms to the World Medical Association Declaration of Helsinki.

### Outcome measures of disease severity

Spirometry was performed as a part of the QMG [10]. The QMG is a 13-item, clinician-administered scale that evaluates strength and endurance in the extremities, neck, ocular, bulbar, and respiratory muscles with a total score range of 0–39 (higher scores indicate greater severity). Of 13 items, one is about respiration (spirometry).

Spirometry was performed in a sitting position using a handheld device, a Vitalograph® model 6300 micro, following the ATS guidelines [11]. Participants were instructed to take a deep breath and then perform a maximal forced expiration. The patients were allowed to hold the spirometer themselves, and the clinician provided vigorous coaching to the participants to empty their lungs. A minimum of three and a maximum of five attempts were performed, depending on the test quality determined by the device. The best results for the forced vital capacity (FVC) and forced expiratory volume in one second (FEV1) were recorded, both in liters and as a percentage of predicted values. The percent of predicted values was automatically calculated by the spirometer, based on age, sex, height, and ethnicity, following the GLI 2012 references [12]. According to the QMG, an FVC ≥ 80% of predicted values is considered normal, while decreasing values corresponded to mild (65–79%), moderate (50–64%), and severe (< 50%) respiratory impairment.

The MG-ADL was used as a patient-reported measure of disease severity [13]. The MG-ADL is an 8-item, clinician-directed but patient-reported questionnaire assessing common MG symptoms over the preceding seven days (score range 0–24, higher scores indicate greater burden). A total score below 3 reflects well-controlled disease, and minimal symptom expression corresponds to a score of 0–1 [14]. One item specifically addresses respiratory function. Patients are asked if they have experienced any respiratory involvement due to MG during the last 7 days. The respiratory item (item 4) ranges from normal (score 0), shortness of breath with exertion (score 1), shortness of breath at rest (score 2), to ventilator dependency (score 3).

Finally, we used the MGC [15]. This scale is a 10-item instrument combining patient history and physical examination, scored on a 4-point ordinal scale (total score range 0–56, higher scores indicate more severe disease). The MGC contains one respiratory item, and this item is worded as the respiratory item of the MG-ADL but is scored differently. The respiratory symptom ranges from normal (score 0), shortness of breath with exertion (score 2), shortness of breath at rest (score 4), to ventilator dependency (score 9).

### Statistical analyses

Continuous variables were presented by mean ± standard deviation (SD) for normally distributed data and medians and interquartile range (IQR) for non-normally distributed data. Normality was assessed visually using histograms and boxplots. Categorical variables were summarized as numbers (*n*) and percentages (%).

Changes over time were analyzed using a paired *t*-test for normally distributed continuous variables, and a Wilcoxon signed rank test for non-normally distributed variables. The correlation between predicted FVC and the MG-ADL respiratory item was assessed using Spearman’s rank correlation coefficient. ANOVA was used for comparing groups.

All tests were two-tailed, and *p*-values ≤ 0.05 were considered statistically significant. All analyses were performed using SAS Enterprise Guide 8.4.

## Results

**Fig. 1:**
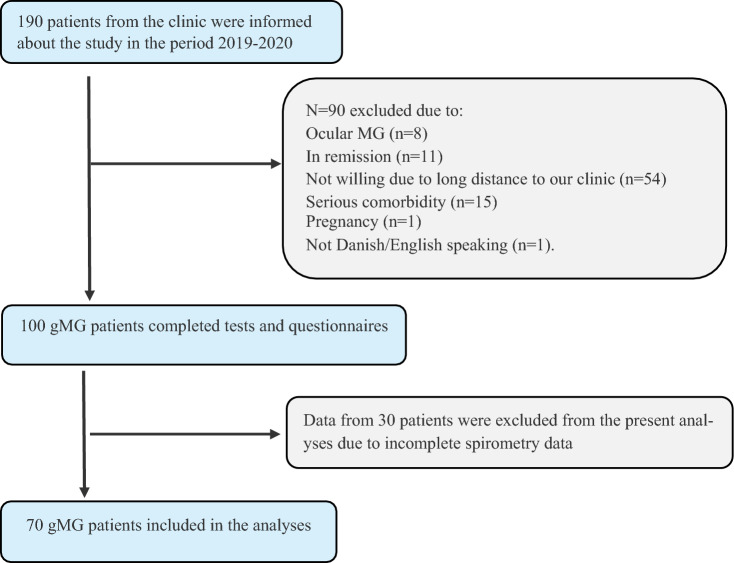
Flowchart

From the outpatient clinic, 190 patients with gMG were informed about the study, and 100 completed tests and questionnaires [9]. For 30 patients, data regarding respiration were missing partially, e.g., FVC in Liters, and to limit the number of missing data, these participants were excluded from the present analyses (Fig. 1).

The 30 participants who were not included (mean age, 62.6 years; 53% women) had test results comparable to those included, with a median MG-ADL score of 2, a median MGC score of 5.5, and a reported median score of 0 in both the MG-ADL and MGC respiratory items.

Baseline characteristics and test results for the 70 included participants are presented in Table 1.

**Table 1:** Characteristics and test results of the included patients

	Minimum	Maximum	Mean (SD)	Median (IQR)	n (%)
Age	19	86	59.2 (16.3)		
Sex, female					41 (58.6)
BMI	15.9	42.6	27.8 (5.9)		
MG-ADL total score	0	17		3 (1–5)	
MG-ADL respiratory item	0	2		0 (0–1)	
MGC total score*	0	36		5 (3–12)	
MGC respiratory item*	0	4		0 (0–2)	
QMG total score	1	21		9 (5–11)	
FVC_L	1.2	6.4	3.1 (1.1)		
FVC predicted (%)	42	170	89.0 (23.7)		
FVC predicted (%) ≥ 80 (n = 45)	81	170	102.3 (17.4)		
FEV1_L	0.96	4.8	2.5 (0.88)		
FEV1 predicted (%)	40	149	84.9 (22.6)		

Legend Table 1: N = 70. *Breathing item missing for 1 person

BMI: Body Mass Index, MG-ADL: The Myasthenia Gravis Activities of Daily Living profile, MGC: Myasthenia Gravis Composite scale, QMG: Quantitative Myasthenia Gravis score, FVC_L: Forced Vital Capacity measured in liters, FVC predicted: Forced Vital Capacity measured as percent of predicted, FEV1_L: Forced Expiratory Volume in 1 s measured in liters, FEV1 predicted: Forced Expiratory Volume in 1 s measured as percent of predicted, SD: standard deviation, IQR: Inter quartile ranges

The mean age of the participants was 59 years, 59% were women, and the overall severity of MG disease was mild to moderate. Of the patients included, 57% reported normal respiration, 36% mild symptoms, and 7% moderate symptoms according to the MG-ADL respiratory item. No participants reported severe symptoms.

Forty-five participants had a predicted FVC ≥ 80%. These patients had corresponding lower MG severity scores (MG-ADL median 2 [IQR 0–5], QMG median 7 [IQR 5–10], MGC median 4 [IQR 2–9]) with median scores of 0 on both the MG-ADL and MGC respiratory items.

There was a large range in the predicted values for FVC and FEV1 (40–170%). For the very low and high measurements, we found consistent results between FVC and FEV1, which were reproduced in patients who were tested twice. The spirometry results were not necessarily reflected in the patients' MG-ADL answers; for example, some patients who had spirometry results showing a low percentage of predicted FVC, e.g., 40%, reported only mild respiratory symptoms in the MG-ADL.

To further characterize participants with respiratory symptoms, two subsamples were defined in the statistical analyses: Subsample A (*n* = 25), consisting of participants with a score > 0 on the respiratory items of the MG-ADL *and* the MGC, and Subsample B (*n* = 25), consisting of participants with a predicted FVC < 80% (Table 2).

**Table 2:** Subsample of patients with respiratory symptoms

	Subsample A (n = 25) Respiratory items > 0		Subsample B (n = 25) Predicted FVC < 80%	
	Mean (SD)	Median (IQR)	Mean (SD)	Median (IQR)
Age	60.5 (15.1)		62.0 (17.4)	
BMI	27.8 (6.6)		27.8 (4.9)	
MG-ADL		4 (3–7)		4 (2–5)
MG-ADL breathing item		1 (1–1)		1 (0–1)
MGC		9 (5–13)		9 (5–13)
MGC breathing item		2 (2–2)		2 (0–2)
QMG		11 (9–13)		11 (9–13)
FVC_L	2.8 (1.0)		2.4 (0.7)	
FVC predicted (%)	82.4 (20.5)		64.9 (10.9)	
FEV1_L	2.2 (0.9)		1.8 (0.5)	
FEV1 predicted (%)	77.0 (22.1)		63.7 (12.9)	

Legend Table 2: BMI: Body Mass Index, MG-ADL: The Myasthenia Gravis Activities of Daily Living profile, MGC: Myasthenia Gravis Composite scale, QMG: Quantitative Myasthenia Gravis score, FVC_L: Forced Vital Capacity measured in liters, FVC predicted: Forced Vital Capacity measured as percent of predicted, FEV1_L: Forced Expiratory Volume in 1 s measured in liters, FEV1 predicted: Forced Expiratory Volume in 1 s measured as percent of predicted, SD: standard deviation, IQR: Inter quartile ranges

The overlap between the two subsamples was 48%, and by coincidence, they included the same number of patients.

In Subsample A, patient responses were identical across the respiratory items of the MG-ADL and MGC (wording identical but scored differently). One patient, however, reported more severe respiratory symptoms on the MG-ADL than on the MGC (dyspnoea at rest versus during physical activity), probably because the scales were completed separately for this patient. Both subsamples had higher median scores on the MG-ADL, MGC, and QMG than the overall sample, indicating more severe MG symptoms.

When applying a broader definition of respiratory symptoms, defined as a score > 0 in either the MG-ADL *or* the MGC respiratory items (*n* = 34), the test results and demographics were like Subsample A, although predicted FVC and FEV1 values were slightly higher (FVC: 85.1%, FEV1: 80.6%).

The cut-off point of 80 in the %predicted FVC was chosen due to the guidelines of the QMG [10].

**Fig. 2:**
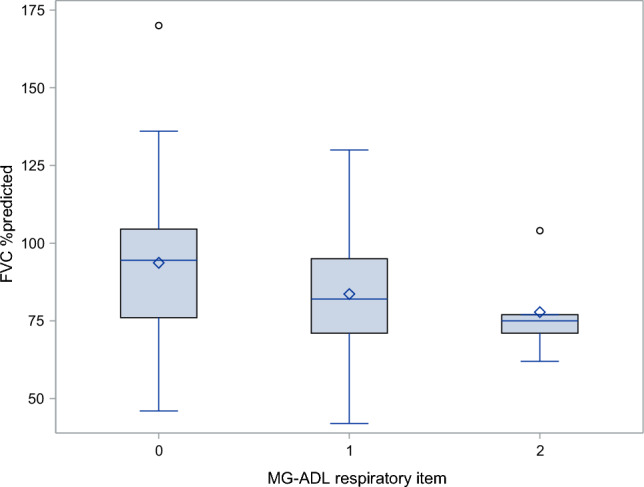
Distribution of the FVC %predicted across the MG-ADL respiratory item in 70 patients with gMG

The distribution of the %predicted FVC across the MG-ADL respiratory item is shown in Fig. 2. The correlation between self-reports and objective measures was weak (*r* = − 0.24, *p* = 0.048, confidence interval −0.45; −0.0004). In addition, when comparing mean %predicted FVC and mean FVC in liters between categories of the MG-ADL respiratory item (%predicted *p* = 0.138, FVC_L *p* = 0.225) or the MG-ADL total score (%predicted *p* = 0.763, FVC_L *p* = 0.812), the findings were not significant, indicating no differences.

A subgroup of 25 participants underwent spirometry twice, with a re-test period of 3 years (Table 3). These 25 patients were a part of a tested sample of 178 patients, and were similar to the 178 patients regarding demographics and disease severity [3].

**Table 3:** Subgroup of patients completing the tests and the spirometry twice

	First spirometry	Second spirometry	Difference Mean (SD)	*P*- value
Age	53.6 (17.3)			
Sex, female	15 (60.0%)			
BMI	27.8 (6.5)	28.2 (6.9)	0.37 (3.0)	.545
MG-ADL	3 (0–6)	2 (0–4)		.302
MG-ADL respiratory item	0 (0–1)	0 (0–1)		.613
MGC *	6 (3–10)	2 (0–5)		.0004
MGC respiratory item*	0 (0–2)	0 (0–1)		.781
QMG	8 (6–11)	7 (4–12)		.083
FVC predicted	86.2 (19.5)	98.5 (16.1)	12.3 (11.1)	<.0001

Legend Table 3: N = 25. *N = 24 due to 1 missing MGC breathing item. BMI: Body Mass Index, MG-ADL: The Myasthenia Gravis Activities of Daily Living profile, MGC: Myasthenia Gravis Composite scale, QMG: Quantitative Myasthenia Gravis score, FVC predicted: Forced Vital Capacity measured as percent of predicted. Age, BMI, and FVC are presented in mean (standard deviation). MG-ADL, MGC, and QMG are presented as median (interquartile ranges). Sex is presented as a number (percentage)

As shown in Table 3, the mean predicted FVC improved from the first to the second assessment. No significant changes were observed in the MG-ADL or QMG scores over the two visits. However, improvements were seen in the MGC. These findings suggest that respiratory function improved over time, despite overall disease severity remaining largely stable.

## Discussion

In this cohort, a relatively large proportion of patients reported respiratory symptoms; however, no severe symptoms (requiring ventilation) were reported, and none of the included participants were in acute respiratory crisis due to MG. Some had previously experienced a crisis but were stable at the time of testing.

We aimed to assess respiratory status in a Danish outpatient clinic, where patients are generally believed to be well-treated (the median total score of the MG-ADL was 3 for the overall cohort). However, despite this expectation, 43% of the patients reported respiratory symptoms, and 25 patients had a FVC below 80% of predicted. This emphasizes the importance of assessing respiratory function, also in an outpatient clinic.

Across different treatment regimens, our findings were very close to the findings from a Canadian outpatient clinic [5] (predicted FVC, 89.0% versus 89.68%, abnormal seated FVC < 80%, 35.7% versus 33.1%). Similar findings were seen in a comparable Brazilian cohort [6], confirming that respiratory insufficiency can be present even in clinically stable patients.

Our findings indicate that the MG-ADL is not a reliable standalone tool for identifying respiratory involvement in MG in an outpatient setting. As illustrated in Fig. 2, there was a wide variation in %predicted FVC values for each response category of the MG-ADL respiratory item, and the correlation with spirometry was weak. These results are in line with previous studies finding poor or insignificant correlates between patient-reported outcomes (e.g., the Myasthenia gravis quality of life scale, MG-QoL15, the myasthenia gravis impairment index, MGII) and FVC [5, 7]. If spirometry is considered the gold-standard objective measure, the MG-ADL does not demonstrate sufficient validity as a standalone screening instrument for respiratory function. However, the narrow ranges and the floor/ceiling effect (57% had a score of 0, and no patients had a score of 3) of the ordinal MG-ADL scale limit the room for finding associations. The correlations might have been stronger if the number of answer possibilities of the MG-ADL respiratory item had been higher. In addition, as the MG-ADL presents the patient's own history of symptoms, there is a risk that the reported respiratory symptoms are due to other issues than MG, possibly explaining some of the weak correlation with spirometry. However, as the confidence interval of the correlation is wide and the *p*-value is borderline significant, the result is uncertain, and conclusions must be interpreted with caution. In addition, as this study is a secondary analysis of previously collected data, no sample estimates are performed, and the study may be underpowered to detect real correlations.

The limited validity of the MG-ADL as a standalone screening tool might be due to limitations of its respiratory item. In contrast, other patient-reported respiratory scales might be more suitable, e.g., the modified Medical Research Council dyspnoea scale [7]. In addition, other measurements, such as the single breath count test, maximal inspiratory pressure (MIP), peak expiratory flow (PEF), and the sniff nasal inspiratory pressure (SNIP), have been found to correlate with FVC in patients with MG [5–7, 16]. The study by Alcantara et al. found that MIPs and SNIPs were sensitive to detecting respiratory dysfunction in MG, and better correlated with disability and quality of life than the FVC [5].

Over time, patients appeared to improve their respiratory function (Table 3). This is encouraging, as it suggests that those with potentially life-threatening symptoms were identified and responded well to treatment. The only other significant improvement observed was the MGC score, combining patient-reported information and clinician-assessed disease severity. Importantly, the respiratory item in the MGC is integrated within a broader clinical evaluation, e.g., strength in neck muscles, which may explain its greater sensitivity to change over time. In this sense, the MGC might bridge the gap between patient experience and objective assessment, making it a potentially valuable tool for identifying and monitoring respiratory involvement in clinical practice [17].

We found a large variation in the percent of predicted values for both FVC and FEV1, ranging from 40 to 170%. However, there were no indications that this variation was due to measurement errors. From a methodological perspective, we decided to keep these values in the analyses to maintain transparency and minimize bias in the study results.

Interestingly, FEV1 (% predicted) values were consistently lower than FVC (% predicted) across the cohort, indicating relatively greater limitation in expiratory flow compared to vital capacity. This may reflect what we have observed in our clinic, that the forced expiratory volume (FEV1) is more sensitive to weakness in the respiratory muscles and the facial muscles used to form a tight seal around the mouthpiece of the spirometer. This observation may warrant further investigation in patients with more active disease or during longitudinal monitoring.

The study was strengthened by the relatively large cohort of well-characterized patients with confirmed gMG, all assessed in a specialized neuromuscular centre using standardized outcome measures. Evaluations were conducted by experienced clinicians trained together to ensure consistency. The availability of a longitudinal subsample further strengthens the reliability of the findings. In addition, one of the authors, an experienced nurse and researcher familiar with living with gMG, contributed valuable insights into the patient perspective throughout the writing process.

Limitations include the primarily cross-sectional design and the limited number of participants in the longitudinal subsample. Generalizability might be limited by the inclusion from a single specialized centre, and the exclusion of patients in acute respiratory crisis further restricts the applicability to more severe cases.

Based on this study of stable outpatients, the clinical implication for practice is that patients' self-reports, along with the MG-ADL, are insufficient for identifying respiratory involvement. Spirometry remains essential, despite being more time-consuming and resource-intensive than the MG-ADL or MGC evaluations. If the clinician suspects respiratory insufficiency, it must be assessed by spirometry, even though respiratory involvement is not recognized by the patient. However, if a screening tool is needed, the MGC, which integrates both patient-reported and clinician-assessed components, might be more appropriate than the MG-ADL. Awareness of respiratory symptoms in the outpatient clinic might prevent disease deterioration where ventilation is needed.

## Conclusion

Findings from this study emphasize that respiratory involvement in stable MG is relatively common. When respiratory impairment is suspected, spirometry remains essential for accurate assessment. Relying solely on the MG-ADL respiratory item may lead to under-detection of respiratory dysfunction, whereas the MGC, which integrates both patient-reported and clinician-assessed components, may offer a more balanced screening approach in clinical practice.

## Data Availability

The datasets used and analyzed during the current study are available from the corresponding author on reasonable request.

## References

[1] Gilhus NE, Andersen H, Andersen LK, Boldingh M, Laakso S, Leopoldsdottir MO et al (2024) Generalized myasthenia gravis with acetylcholine receptor antibodies: a guidance for treatment. Eur J Neurol 31(5):e16229. 10.1111/ene.16229 38321574 10.1111/ene.16229 PMC11236053

[2] Hansen JS, Danielsen DH, Somnier FE, Frøslev T, Jakobsen J, Johnsen SP et al (2016) Mortality in myasthenia gravis: a nationwide population-based follow-up study in Denmark. Muscle Nerve 53(1):73–77. 10.1002/mus.24697 25914186 10.1002/mus.24697

[3] Andersen LK, Petersen KG, Deurell EM, Lagoni A, Kaalund AB, Flensted IF et al (2025) EQ-5D-5L is a relevant tool for detecting patients with myasthenia gravis needing medical treatment. J Neurol Sci 473:123493. 10.1016/j.jns.2025.123493 40233650 10.1016/j.jns.2025.123493

[4] Gilhus NE (2023) Myasthenia gravis, respiratory function, and respiratory tract disease. J Neurol 270(7):3329–3340. 10.1007/s00415-023-11733-y 37101094 10.1007/s00415-023-11733-y PMC10132430

[5] Alcantara M, Barnett-Tapia C, Bril V, Mannan S, Shabanpour J, Riaz S et al (2024) Office-based respiratory assessment in patients with generalized myasthenia gravis. Neuromuscul Disord 40:1–6. 10.1016/j.nmd.2024.05.005 38776756 10.1016/j.nmd.2024.05.005

[6] Oliveira EF, Nacif SR, Urbano JJ, Silva AS, Oliveira CS, Perez EA et al (2017) Sleep, lung function, and quality of life in patients with myasthenia gravis: a cross-sectional study. Neuromuscul Disord NMD 27(2):120–127. 10.1016/j.nmd.2016.11.015 28062220 10.1016/j.nmd.2016.11.015

[7] Aguirre F, Fernández RN, Arrejoría RM, Manin A, Cores VE, Sivori M et al (2023) Peak expiratory flow and the single-breath count test as markers of respiratory function in patients with myasthenia gravis. Neurol Engl Ed 38(6):405–411. 10.1016/j.nrleng.2020.09.006 10.1016/j.nrleng.2020.09.006 35842128

[8] Teeselink S, Vincenten SCC, Voermans NC, Groothuis JT, Doorduin J, Wijkstra PJ et al (2022) Long-term follow-up of respiratory function in facioscapulohumeral muscular dystrophy. J Neurol 269(7):3682–3689. 10.1007/s00415-022-10990-7 35147730 10.1007/s00415-022-10990-7 PMC8831680

[9] Andersen LK, Jakobsson AS, Revsbech KL, Vissing J (2022) Causes of symptom dissatisfaction in patients with generalized myasthenia gravis. J Neurol 269(6):3086–3093. 10.1007/s00415-021-10902-1 34806129 10.1007/s00415-021-10902-1

[10] Tindall RS, Rollins JA, Phillips JT, Greenlee RG, Wells L, Belendiuk G (1987) Preliminary results of a double-blind, randomized, placebo-controlled trial of cyclosporine in myasthenia gravis. N Engl J Med 316(12):719–724. 10.1056/NEJM198703193161205 3547126 10.1056/NEJM198703193161205

[11] Graham BL, Steenbruggen I, Miller MR, Barjaktarevic IZ, Cooper BG, Hall GL et al (2019) Standardization of spirometry 2019 update. An official American Thoracic Society and European Respiratory Society technical statement. Am J Respir Crit Care Med 200(8):e70-88. 10.1164/rccm.201908-1590ST 31613151 10.1164/rccm.201908-1590ST PMC6794117

[12] Quanjer PH, Stanojevic S, Cole TJ, Baur X, Hall GL, Culver BH et al (2012) Multi-ethnic reference values for spirometry for the 3-95-yr age range: the global lung function 2012 equations. Eur Respir J 40(6):1324–1343. 10.1183/09031936.00080312 22743675 10.1183/09031936.00080312 PMC3786581

[13] Wolfe GI, Herbelin L, Nations SP, Foster B, Bryan WW, Barohn RJ (1999) Myasthenia gravis activities of daily living profile. Neurology 52(7):1487–1489 10227640 10.1212/wnl.52.7.1487

[14] Vissing J, Jacob S, Fujita KP, O’Brien F, Howard JF, REGAIN study group (2020) “Minimal symptom expression” in patients with acetylcholine receptor antibody-positive refractory generalized myasthenia gravis treated with eculizumab. J Neurol 267(7):1991–2001. 10.1007/s00415-020-09770-y 32189108 10.1007/s00415-020-09770-y PMC7320935

[15] Burns TM, Conaway MR, Cutter GR, Sanders DB, Muscle Study Group (2008) Construction of an efficient evaluative instrument for myasthenia gravis: the MG composite. Muscle Nerve 38(6):1553–1562. 10.1002/mus.21185 19016543 10.1002/mus.21185

[16] Elsheikh B, Arnold WD, Gharibshahi S, Reynolds J, Freimer M, Kissel JT (2016) Correlation of single-breath count test and neck flexor muscle strength with spirometry in myasthenia gravis. Muscle Nerve 53(1):134–136. 10.1002/mus.24929 26437790 10.1002/mus.24929 PMC4715713

[17] Burns TM (2012) The MG composite: an outcome measure for myasthenia gravis for use in clinical trials and everyday practice. Ann N Y Acad Sci 1274:99–106. 10.1111/j.1749-6632.2012.06812.x 23252903 10.1111/j.1749-6632.2012.06812.x

